# Amino Acid Carbamates As Prodrugs Of Resveratrol

**DOI:** 10.1038/srep15216

**Published:** 2015-10-14

**Authors:** Andrea Mattarei, Michele Azzolini, Martina La Spina, Mario Zoratti, Cristina Paradisi, Lucia Biasutto

**Affiliations:** 1 University of Padova, Department of Chemical Sciences, Via F. Marzolo 1, 35131 Padova, Italy; 2 CNR Neuroscience Institute, Viale G. Colombo 3, 35121 Padova, Italy; 3 University of Padova, Department of Biomedical Sciences, Viale G. Colombo 3, 35121 Padova, Italy; 4 NÓOS srl, via Campello sul Clitunno 34, 00181 Roma, Italy

## Abstract

Resveratrol (3, 5, 4′-trihydroxy-*trans*-stilbene), a plant polyphenol, has important drug-like properties, but its pharmacological exploitation *in vivo* is hindered by its rapid transformation via phase II conjugative metabolism. One approach to bypass this problem relies on prodrugs. We report here the synthesis, characterization, stability and *in vivo* pharmacokinetic behaviour of prodrugs of resveratrol in which the OH groups are engaged in an N-monosubstituted carbamate ester (-OC(O)NHR) linkage with a natural amino acid (Leu, Ile, Phe, Thr) to prevent conjugation and modulate the physicochemical properties of the molecule. We also report a convenient, high-yield protocol to obtain derivatives of this type. The new carbamate ester derivatives are stable at pH 1, while they undergo slow hydrolysis at physiological pH and hydrolyse with kinetics suitable for use in prodrugs in whole blood. After administration to rats by oral gavage the isoleucine-containing prodrug was significantly absorbed, and was present in the bloodstream as non-metabolized unaltered or partially deprotected species, demonstrating effective shielding from first-pass metabolism. We conclude that prodrugs based on the N-monosubstituted carbamate ester bond have the appropriate stability profile for the systemic delivery of phenolic compounds.

Resveratrol (trans-3,5,4′-trihydroxystilbene), is a naturally occurring phytoalexin produced by some plants (e.g. *V. vinifera*) in response to stresses^1^. It exhibits a range of activities of biomedical interest, including lifespan extension in model systems^2^, protection of the cardiovascular apparatus^3^,^4^,^5^,^6^, anti-inflammatory activity^3^, improvement of glucose handling in diabetes^7^,^8^, decrease of fat and cholesterol load^9^,^10^,^11^,^12^, improvements of functionality in aging^2^,^13^,^14^,^15^, neuroprotection^16^, cancer chemoprevention^17^,^18^ and potentiation of chemotherapy^19^,^20^.

The efficacy of orally administered resveratrol depends on its absorption, metabolism, and tissue distribution. Clinical studies with humans reported mostly small effects, and sometimes controversial results^21^,^22^. Beneficial effects *in vivo* are in fact limited by low bioavailability. Thus, for example, administration of even very large doses (2.5–5.0 g) to volunteers resulted in circulating levels of non-metabolized resveratrol so low as to be considered insufficient for any bioactivity^23^. Resveratrol, like other polyphenols, is rapidly converted to phase II metabolites (mainly glucuronides and sulfates) during absorption and first pass through the liver. These hydrophilic metabolites are re-exported to the intestinal lumen by enterocyte ABC transporters, and/or rapidly excreted with faeces and urine^23^,^24^. Nonetheless, given their preponderance, their bioactivities are now under investigation to verify whether they may account for resveratrol’s effects. The available data suggest that a level of activity may well be maintained in specific contexts (e.g.^25^,^26^,^27^,^28^), but conjugation often determines at least a partial loss of bioefficacy^29^,^30^,^31^,^32^,^33^,^34^. Retention of bioactivity *in vivo* may be due in part to the metabolites acting as a temporary reservoir from which the more active species may be regenerated through the activity of enzymes such as glucuronidases and sulfatases^35^,^36^. While investigation of these aspects continues, a reduction or delay of phase II metabolism remains a primary target for the biomedical exploitation of resveratrol.

One of the strategies used to prevent drug metabolism and enhance bioavailability and effectiveness is based on prodrugs. In a successful resveratrol prodrug, the natural active compound is protected against phase II metabolism by having the reactive hydroxyl groups masked as suitably reversible derivatives (-OH → -OXR). The capped hydroxyl functionalities (-OXR) should withstand the processes of absorption, first pass through the liver and distribution. Once in the bloodstream, the prodrug should then release the active natural compound, resveratrol (-OXR → -OH). Appropriate regeneration kinetics may provide a form of sustained delivery, a possibly convenient alternative to other approaches such as slow-release capsules. Optimal prodrug performance is sought by modulating its physicochemical properties using the best combination of X, the reversible protecting linkage (ester, carbamate, acetal, …), and of R, the promoiety. The type of linkage X is mostly relevant for prodrug persistence whereas the promoiety R may determine its solubility in aqueous media and partitioning between aqueous and lipidic (membrane) phases. One crucial additional requirement is that hydrolysis of the prodrug produce, besides the original active natural compound, safe by-products.

The carbamoyl moiety provides a versatile protecting group for phenols (-OH → -OC( = O)NR′R″)^37^,^38^, which allows fine-tuning of the properties of the molecule since the two substituents on nitrogen, R′ and R″, can be tailored to modulate the stability and physicochemical properties of the resulting prodrug. Several prodrugs have been developed to improve oral bioavailability of phenolic compounds^39^,^40^,^41^ but surprisingly there are only few examples of carbamate ester prodrugs of polyphenols. Mulholland *et al.*^42^ reported a water-soluble glycine N-monosubstituted carbamate ester prodrug (QC12) of the flavonoid quercetin. The advantage of QC12 was its high aqueous solubility. Quercetin-amino acid N-monosubstituted carbamate esters were also synthesized by Kim *et al.*^43^ and showed remarkable increases in water solubility, stability, and cell permeability compared with the parent polyphenol. We have previously reported that N,N-disubstituted carbamoyl derivatives of resveratrol are too stable under physiological conditions to be of practical use as prodrugs^44^. We therefore turned our attention to N-monosubstituted carbamoyl derivatives -OC( = O)NHR and report here the synthesis and the properties of a family of new prodrugs in which three units of a natural amino acid are linked to resveratrol via carbamoyl derivatization of the three hydroxyl groups (Fig. 1).

Use of amino acids as promoieties in prodrug design has been recently reviewed^45^. Their presence increases water solubility, and they are considered to be safe hydrolysis by-products. Moreover, we reasoned that prodrugs incorporating suitable amino acids in the promoieties might be recognized and transported by the various epithelial amino acid^46^ and peptide^47^,^48^,^49^ carrier systems. We selected Leu, Ile and Phe (Fig. 1) because intestinal epithelia are well provided with carriers of neutral/large amino acids. These are, in particular, known substrates of the LAT (Large-neutral Amino acid Transporters or L-type Amino acid Transporters)^50^ systems, with affinities in the μM range. Amino acid transporters LAT1 and LAT2 have been previously exploited for the transport/absorption of drugs; they are especially useful for the permeation of the blood brain barrier^51^,^52^,^53^. We also included threonine as a “control” amino acid because it is not efficiently transported by LAT (although it is a known substrate of the ASCT2 system)^46^.

The performance of the new derivatives **2**–**5** was assessed by kinetic studies of hydrolysis in aqueous media under different pH conditions of physiological relevance (strongly acidic, as in the stomach, and near neutral, as in the intestine) as well as in blood. Pharmacokinetic experiments in rats were also performed in order to evaluate *in vivo* absorption, stability and metabolism of these new prodrugs.

## Results & Discussion

### Synthesis

**Figure 2:**
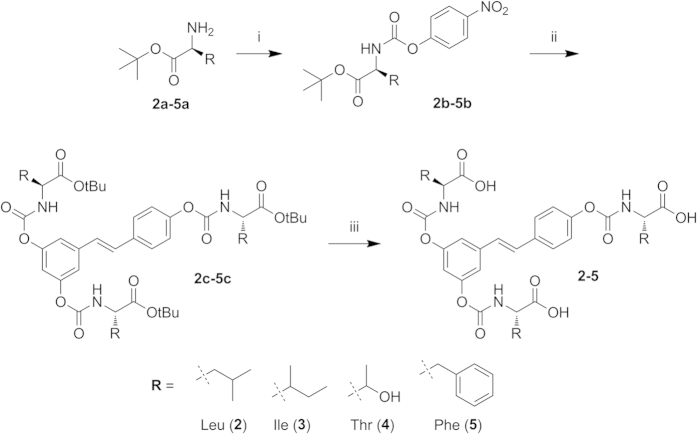
Synthesis of resveratrol N-monosubstituted carbamate ester prodrugs. *Reagents and conditions*: (i) bis-(4-nitrophenyl) carbonate, DMAP, ACN, 50 °C, 3 h; (ii) resveratrol (1), ACN, DMAP, 50 °C, 24 h; (iii) DCM/TFA 1:1, TIPS, from 0 °C to r.t., 1.5 h.

Synthesis of N-monosubstituted carbamate esters is usually carried out in two steps: reaction of the desired primary amine with phosgene or its equivalent to give a reactive isocyanate derivative, followed by coupling of this intermediate with the phenolic function^54^. These procedures, however, led to the desired trisubstituted resveratrol derivatives in low yields, probably due to the high reactivity of the isocyanate group promoting side reactions of polymerization entraining the stilbene C-C double bond. In this study, therefore, we utilized and optimized an alternative approach via an activated 4-nitrophenyl carbamate intermediate^43^. By this route, outlined in Fig. 2, we prepared N-monosubstituted resveratrol carbamate esters **2**–**5** in fair overall yields.

The procedure involves conversion of the C-protected amino acids **2a**–**5a** into the corresponding activated 4-nitrophenyl carbamates **2b**–**5b**. After isolation, these intermediates are treated with resveratrol to afford the product of transesterification, the *t*-butyl protected resveratrol amino acid carbamate ester conjugates **2c**–**5c** in good to excellent yields under mild conditions. The desired final products **2**–**5** are readily obtained by removal of the *t*-butyl protecting group by treatment with TFA.

### Hydrolysis studies

The hydrolytic reactivity of the new derivatives **2**–**5** was tested in aqueous solutions mimicking gastric and intestinal pH and also in blood. All compounds turned out to be highly stable at pH values close to that of the human stomach, no reaction occurring over 24 hours at 37 °C in 0.1 N HCl, and they underwent slow hydrolysis at near-neutral pH (pH 6.8, representing intestinal pH) thus ensuring protection of the phenolic moieties from first pass metabolism during absorption in the gastrointestinal tract. In contrast all the synthesized prodrugs hydrolyzed in murine whole blood, with kinetics suitable for use as prodrugs.

**Figure 3:**
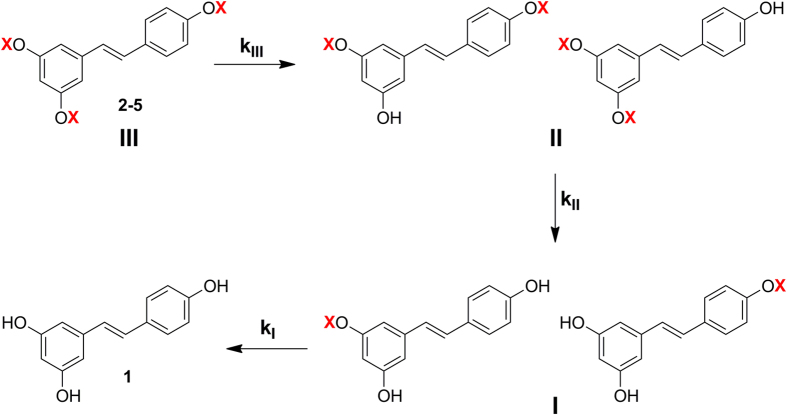
Stepwise hydrolysis of 2–5 to resveratrol (1) and simplified kinetic treatment.

Hydrolysis of derivatives **2**–**5** to resveratrol proceeds stepwise through intermediates in which initially one and then two protecting groups have been sequentially removed, as shown in Fig. 3. There are two possible isomeric disubstituted intermediates and, likewise, two monosubstituted intermediates. By HPLC-UV and HPLC-MS analyses in most cases we were able to detect and separate all of them. However, to simplify the kinetic analysis of the data we considered each pair of isomeric intermediates, resulting from the first and second hydrolysis steps, as a single species, i.e. the two disubstituted and the two monosubstituted intermediates were handled as species **II** and **I**, respectively (Fig. 3). Fitting of the experimental data was then performed considering consecutive pseudo first order reactions and using a set of equations analogous to those described by Kozerski *et al.*^55^

**Figure 4:**
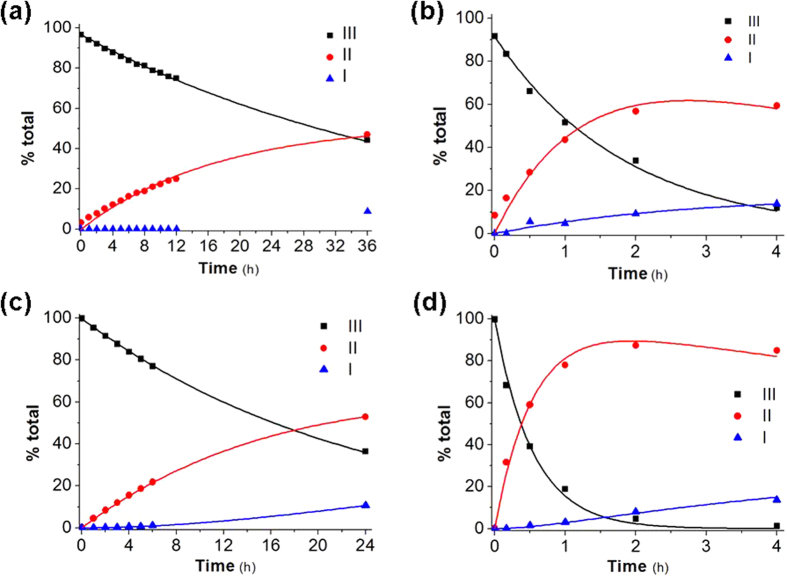
Kinetics and products of hydrolysis of derivatives 3 (a,b) and 5 (c,d): (a) and (c): aqueous PBS 0.1 M at pH 6.8 and 37 °C; (b) and (d): rat blood. The experimental data were processed and fitted as described in the text, with reference to Fig. 3. Please note that the abscissa scale varies among panels.

Examples are shown in Fig. 4, which reports the results of kinetic and product analysis for hydrolysis of derivatives **3** (a, b) and **5** (c, d) in aqueous PBS 0.1 M, at pH 6.8 and 37 °C (a, c) and in rat blood (b, d).

**Figure 5:**
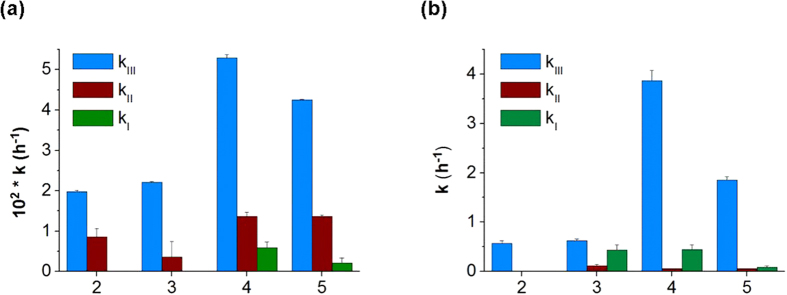
Observed pseudo first order rate constant for hydrolysis of resveratrol derivatives: (a) PBS 0.1 M, pH 6.8, 37 °C, (b) rat blood. Please note the different ordinate scales in (**a**,**b**).

The full set of kinetic results obtained for hydrolysis of derivatives **2**–**5** according to the procedures described above is presented in Table 1 and, graphically, in Fig. 5.

**Figure 6:**
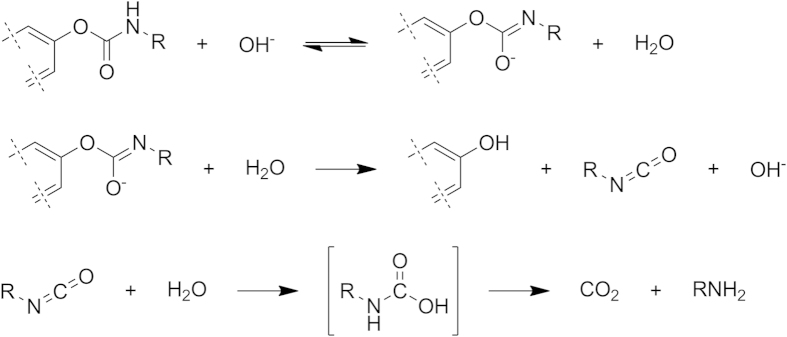
Mechanism of base-induced hydrolysis of carbamates 2–5.

The stability of our derivatives **2**–**5** in acidic solution and their reactivity at higher pH’s are consistent with a mechanism of base catalyzed hydrolysis of carbamates proceeding via deprotonation and elimination to give an isocyanate intermediate which rapidly adds water and decomposes releasing carbon dioxide and the amino acid (Fig. 6)^56^.

The data show that hydrolysis is much faster in blood, suggesting the involvement of enzymes. This notion is reinforced by the variability of the rates from one compound to the other. Hansen^38^ and co-workers have demonstrated that the predominant rate-accelerating component of plasma (human in that study) is albumin.

### Pharmacokinetics studies

Derivatives **2**–**5** were then tested for their *in vivo* absorption and metabolism by performing pharmacokinetic studies after oral administration to rats. Each compound was administered as a single intragastric bolus, in an equimolar dose/kg body weight (88 μmol/kg). Blood samples were taken at different time points over a 24 h period, treated and analyzed as described in the Materials and Methods section. Each experiment was replicated at least 3 times.

**Figure 7:**
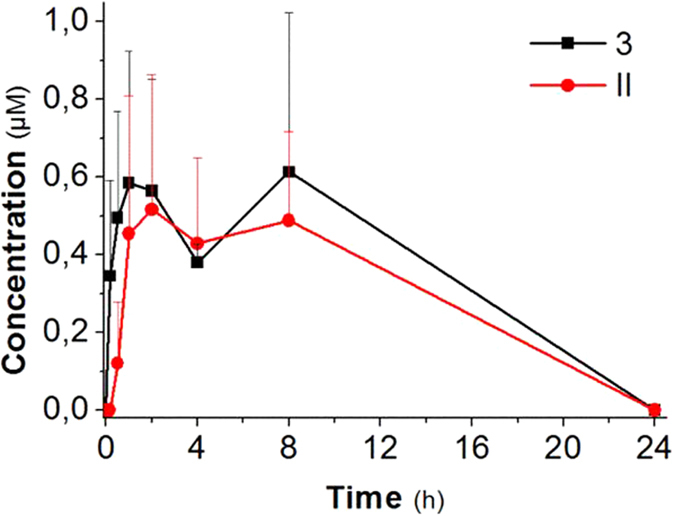
Blood pharmacokinetic profile after oral administration of derivative 3. Data represent average values +s.d. N = 3.

Contrary to our expectations the linkage of resveratrol through the N-monosubstituted carbamate ester to amino acids resulted in poor absorption of the prodrugs after oral administration. We can only speculate that this result is probably due to the excessive hydrophilicity conferred by the three carboxylic ionisable groups present in the derivatives, which hinder permeation of lipophilic biomembranes. It is furthermore evident that these trisubstituted prodrugs were not readily recognized and/or transported by aminoacid carriers. The best results were obtained with the isoleucine derivative, **3**, as summarized in Fig. 7. About one hour after oral administration the concentration of this prodrug in blood was higher than 0.5 μM and remained around this level for several hours (Fig. 7). Disubstituted hydrolysis products (**II**, Fig. 3) were also present, indicating the bioreversibility of the carbamate linker also *in vivo* and, interestingly, their level was about as high as that of their precursor, the trisubstituted derivative **3**. Notably, neither sulfated nor glucuronidated species appeared in the bloodstream confirming the protection by the N-monosubstituted carbamate linker from first pass metabolism during absorption.

The concentration of tri- and di- substituted carbamate-Ile resveratrol prodrugs was substantially maintained for at least 8 hours, i.e. ample time for equilibration with organs. This behaviour may be due to the fact that the derivative is constantly absorbed from the intestinal mucosa and/or that it is slowly cleared from the body. Resveratrol (**1**) and monosubstituted carbamate-Ile (**I**) were not detected in the bloodstream probably because the blood concentration of these species were too low for reliable measurement. Nature and levels of the species found in blood may not be representative of the nature and levels of stilbenoid species in other organs: the hydrophilicity of the amino acid-decorated compounds is expected to result in their enrichment in blood, while less hydrophilic molecules, e.g. resveratrol itself, are expected to associate with membrane-rich compartments^57^,^58^.

In any case, the prodrug appears to perform as a slow-action vehicle providing a sustained delivery of precursor. By comparison, blood concentration of resveratrol after oral administration of an equimolar amount of resveratrol itself peaks at approximately 1 μM after only about 10 minutes from administration, and rapidly declines. Phase II metabolites peak (∼10 μM) at about 1 h, and then decline by 80–90% over the next 7 hours^59^.

In contrast with the behaviour of **3**, administration of compounds **4** and **5** did not result in the appearance in blood samples of detectable amounts of either resveratrol, products of partial hydrolysis (**II** and **I**) or any metabolites. Similar results were also obtained with **2**, although in this case the presence of very small amounts of partially hydrolyzed derivatives (**II** and **I**) cannot be excluded due to interfering co-eluting peaks due to the matrix.

## Conclusions

The N-monosubstituted carbamate bond is a convenient linker for prodrugs of resveratrol^60^ which has been coupled in this study with amino acid promoieties. All synthesized compounds have good solubility and stability properties in aqueous media and in blood for use as resveratrol prodrugs. *In vivo* pharmacokinetic studies revealed an interesting behaviour of the isoleucine derivative **3**, which acts as a slow-action vehicle providing a sustained delivery of the prodrug and partially hydrolysed unconjugated species, indicating protection from first pass metabolism during absorption. However, the choice of amino acid as promoieties proved to be unsatisfactory in terms of absorption of the prodrug, probably due to the excessive hydrophilicity of the resulting prodrugs bearing three ionisable groups, and because carrier-mediated uptake apparently did not take place to a significant extent. Possibly the prodrugs were too large and ramified to be handled by the transporters. As a future development of this work, we aim to seek a better performing promoiety in order to improve absorption.

## Methods

### Chemistry

#### Materials and instrumentation

Resveratrol was purchased from Waseta Int. Trading Co. (Shangai, P.R.China). Other starting materials and reagents were purchased from Aldrich, Fluka, Merck-Novabiochem, Riedel de Haen, J.T. Baker, Cambridge Isotope Laboratories Inc., Acros Organics, Carlo Erba and Prolabo, and were used as received. TLCs were run on silica gel supported on plastic (Macherey-Nagel Polygram^®^SIL G/UV_254_, silica thickness 0.2 mm) and visualized by UV detection. Flash chromatography was performed on silica gel (Macherey-Nagel 60, 230–400 mesh granulometry (0.063–0.040 mm)) under air pressure. The solvents were analytical or synthetic grade and were used without further purification. ^1^H NMR spectra were recorded with a Bruker AC250F spectrometer operating at 250 MHz and a Bruker AVII500 spectrometer operating at 500 MHz. Chemical shifts (δ) are given in ppm relative to the signal of the solvent. HPLC-UV analyses were performed with an Agilent 1290 Infinity LC System (Agilent Technologies), equipped with binary pump and a diode array detector (190–500 nm). HPLC/ESI-MS analyses and mass spectra were performed with a 1100 Series Agilent Technologies system, equipped with binary pump (G1312A) and MSD SL Trap mass spectrometer (G2445D SL) with ESI source. ESI-MS positive spectra of reaction intermediates and final purified products were obtained from solutions in acetonitrile, eluting with a water:acetonitrile, 1:1 mixture containing 0.1% formic acid. High resolution mass measurements were obtained using a Mariner ESI-TOF spectrometer (PerSeptive Biosystems). HPLC-MS analysis was used to confirm purity (>95% in all cases).

### Synthesis of derivatives 2–5

#### General procedure for the preparation of activated 4-nitrophenyl urethanes (**2b-5b**, Fig. 2)

A solution of amino acid t-butyl ester (**2a–5a**) (8.2 mmol, 1.0 eq.) and DMAP (2.00 g, 16.4 mmol, 2.0 eq.) in acetonitrile (15 mL) was added dropwise to a solution of bis(4-nitrophenyl) carbonate (2.74 g, 9.0 mmol, 1.1 eq.) in acetonitrile (15 mL) and the resulting solution was stirred at 50 °C for 3 hours. The reaction mixture was then diluted in DCM (150 mL) and washed with 0.5 N HCl (100 mL). The aqueous layer was washed with DCM (5 × 100 mL) and all the organic fractions were collected, dried over MgSO_4_ and filtered. The solvent was evaporated under reduced pressure and the residue was purified by flash chromatography.

#### Tert-butyl 4-methyl-2-(((4-nitrophenoxy)carbonyl)amino)pentanoate (**2b**)

Purified by flash chromatography using DCM:Acetone:Hexane = 8:0.5:1.5 as eluent. The first two spots were collected and the solvent was evaporated under reduced pressure, then the residue was absorbed on silica and purified by flash chromatography using Hexane:Ethyl Ether = 6.5:3.5 as eluent. 65% yield as a pale yellow oil. ^1^H-NMR (250 MHz, CDCl_3_) δ (ppm): 0.97–1.01 (m, 6 H, 2 ×-CH-C*H*_*3*_), 1.49 (s, 1 H, -C*H*-CH_3_), 1.52–1.85 (m, 11 H, 3 ×-C-C*H*_*3*_and -CH-C*H*_*2*_-), 4.27–4.36 (m, 1 H, NH-C*H*-), 5.56 (d, 1 H, -CH-N*H*-, ^3^J_H-H_ = 8.5 Hz), 7.33 (d, 2 H, Ar-*H*, ^3^J_H-H_ = 9.25 Hz), 8.24 (d, 2 H, Ar-*H*, ^3^J_H-H_ = 9.25 Hz); ^13^C-NMR (62.9 MHz, CDCl_3_) δ (ppm): 171.7, 155.8, 152.7, 125.1, 122.0, 82.5, 53.2, 42.0, 28.0, 27.9, 24.9, 22.8, 22.0. ESI-MS (ion trap): m/z 353 [M+H]^+^; HRMS (ESI+): *m/z* 353.1720 [M+H]^+^, calcd for C_17_H_25_N_2_O_6_: 353.1713.

#### Tert-butyl 3-methyl-2-(((4-nitrophenoxy)carbonyl)amino)pentanoate (**3b**)

Purified by flash chromatography using DCM:Acetone:Hexane = 8:0.5:1.5. 93% yield as a pale yellow oil. ^1^H-NMR (300 MHz, CDCl_3_) δ (ppm): 0.95–1.38 (m, 5 H, -CH_2_-C*H*_*3*_, CH_3_-C*H*_*2*_-), 1.52 (s, 9 H, 3 ×-C-C*H*_*3*_), 1.87–2.04 (m, 1 H, -C*H*-CH_2_-), 4.29 (dd, 1 H, -C*H*-NH-, ^3^J_H-H_ = 8.7, 4.3 Hz), 5.76 (d, 1 H, -CH-N*H*-, ^3^J_H-H_ = 8.6 Hz), 7.32–7.37 (d, 2 H, Ar-*H*, ^3^J_H-H_ = 9.25 Hz), 8.22–8.28 (d, 2 H, Ar-*H*, ^3^J_H-H_ = 9.25 Hz); ^13^C-NMR (300 MHz, CDCl_3_) δ(ppm): 172.2, 157.6, 154.6, 145.5, 126.8, 123.7, 84.3, 79.2, 78.8, 78.3, 60.5, 29.8, 26.9, 17.1, 13.4. ESI-MS (ion trap): m/z 353 [M+H]^+^. ESI-MS (ion trap): m/z 353 [M+H]^+^; HRMS (ESI+): *m/z* 353.1724 [M+H]^+^, calcd for C_17_H_25_N_2_O_6_: 353.1713.

#### Tert-butyl 3-(tert-butoxy)-2-(((4-nitrophenoxy)carbonyl)amino)butanoate (**4b**)

Purified by flash chromatography using DCM:Acetone:Hexane = 8:0.5:1.5 as eluent. 93% yield as a pale yellow oil. ^1^H-NMR (250 MHz, CDCl_3_) δ (ppm): 1.19 (s, 9 H, 3 × C-C*H*_*3*_), 1.28 (d, 3 H, -CH-C*H*_*3*_, ^3^J_H-H_ = 6.3 Hz), 1.49 (s, 9 H, 3 ×-C-C*H*_*3*_), 4.05–4.17 (m, 1 H, -O-C*H*-), 4.22–4.33 (m, 1 H, NH-C*H*-), 5.94 (d, 1 H, CH-N*H*-, ^3^J_H-H_ = 9.5 Hz), 7.35 (d, 2 H, Ar-*H*, ^3^J_H-H_ = 9.25 Hz), 8.27 (d, 2 H, Ar-*H*, ^3^J_H-H_ = 9.25 Hz); ^13^C-NMR (62.9 MHz, CDCl_3_) δ(ppm): 169.7, 156.2, 153.8, 144.9, 125.2, 122.2, 82.5, 74.2, 67.2, 60.8, 28.9, 28.3, 21.4. ESI-MS (ion trap): m/z 397 [M+H]^+^. HRMS (ESI+): *m/z* 397.1977 [M+H]^+^, calcd for C_19_H_29_N_2_O_7_: 397.1975.

#### Tert-butyl 2-(((4-nitrophenoxy)carbonyl)amino)-3-phenylpropanoate (**5b**)

Purified by flash chromatography using DCM:Acetone = 9:1 as eluent. 81% yield as a pale yellow oil. 1H-NMR (250 MHz, CDCl3) δ (ppm): 1.54 (s, 9 H, 3 ×-C-CH_3_), 3.05–3.35 (m, 2 H, 2 × C-CH_2_), 4.70 (dd, 1 H, -NH-C*H*-CH_2_- J = 14.7, 6.5 Hz), 6.33 (d, 1 H, -N*H*-, J = 8.3  Hz), 7.22–7.45 (m, 7 H, Ar-*H*), 8.23 (d, 2 H, Ar-*H*, ^3^J_H-H_ = 15.5 Hz); 13C-NMR (62.9 MHz, CDCl3) δ (ppm): 170.4, 155.8, 152.6, 144.6, 136.0, 129.5, 128.1, 127.5, 124.9, 121.9, 82.7, 55.6, 38.2, 27.3. ESI-MS (ion trap): m/z 387 [M+H]^+^. HRMS (ESI+): *m/z* 387.1547 [M+H]^+^, calcd for C_20_H_23_N_2_O_6_: 387.1556.

#### General procedure for the preparation of 3,4′,5-N-monosubstituted-resveratrol carbamate esters (**2c–5c**)

A solution of resveratrol (0.24 g, 1.1 mmol, 1.0 eq.) and DMAP (0.52 g, 4.2 mmol, 4.0 eq.) in ACN (15 mL) was added to a solution of the activated 4-nitrophenyl urethane (**2b**–**5b**) (4.8 mmol, 4.5 eq) in ACN (5 mL) and the resulting mixture was allowed to react under vigorous stirring at 50 °C for 24 h. The reaction mixture was diluted with DCM (150 mL) and washed with 0.5 N HCl (100 mL). The aqueous layer was washed with DCM (5 × 75 mL) and all the organic fractions were collected, dried over MgSO_4_ and filtered. The solvent was evaporated under reduced pressure and the residue was purified by flash chromatography.

#### (E)-di-tert-butyl 2,2′-((((5-(4-(((1-(tert-butoxy)-4-methyl-1-oxopentan-2-yl)carbamoyl)oxy)styryl)-1,3-phenylene)bis(oxy))bis(carbonyl))bis(azanediyl))bis(4-methylpentanoate) (**2c**)

Purified by flash chromatography using CHCl_3_ as eluent. 55% yield as a colourless oil. ^1^H-NMR (250 MHz, CDCl_3_) δ (ppm): 0.93 (m, 18 H, 6 × CH-C*H*_*3*_), 1.44 (s, 27 H, 9 ×-C-C*H*_*3*_), 1.48–1.53 (m, 9 H, 3 × CH_3_-C*H* and 3 ×-CH-C*H*_*2*_), 4.23–4.32 (m, 3 H, 3 ×-NH-C*H*), 5.64 (m, 3 H, 3 ×-N*H*-), 6.80–7.07 (m, 7 H, H-4, H-3′, H-5′, H-2, H-6, H-7, H-8), 7.47 (d, 2 H, ^3^J_H-H_ = 8.5 Hz, H-2′, H-6′); ^13^C-NMR (62.9 MHz, CDCl_3_) δ (ppm): 172.1, 172.3, 154.0, 153.7, 151.4, 150.5, 139.0, 134.0, 129.2, 134.0, 129.2, 127.3, 127.0, 121.7, 116.4, 114.2, 82.0, 53.1, 41.9, 27.9, 24.8, 22.8, 21.9. ESI-MS (ion trap): m/z 868 [M+H]^+^. HRMS (ESI+): *m/z* 868.4966 [M+H]^+^, calcd for C_47_H_70_N_3_O_12_: 868.4959.

#### (E)-di-tert-butyl 2,2′-((((5-(4-(((1-(tert-butoxy)-3-methyl-1-oxopentan-2-yl)carbamoyl)oxy)styryl)-1,3-phenylene)bis(oxy))bis(carbonyl))bis(azanediyl))bis(3-methylpentanoate) (**3c**)

Purified by flash chromatography using Hexane: EtOAc = 8:2. The first spot was separated and the remaining spots were collected and the solvent was evaporated under reduced pressure, then purified by flash chromatography using DCM:Hexane:EtOAc = 7:2:1. 60% yield as a colourless oil. ^1^H-NMR (300 MHz, CDCl_3_) δ (ppm): 0.87–1.05 (m, 18 H, 3 × CH_2_-C*H*_*3*_, 3 × CH-C*H*_*3*_), 1.12–1.33 (m, 6 H, 3 × CH-C*H*_*2*_-CH_3_), 1.49 (s, 27 H, 9 × C-C*H*_*3*_), 1.87–2.00 (m, 3 H, 3 × CH-CH-CH_3_), 4.11 (q, 1 H, -NH-*CH*-CH-, ^3^J_H-H_ = 7.1 Hz), 4.23–4.33 (m, 2 H, 2 ×-NH-*CH*-CH-), 5.79 (d, 3 H, 3 ×-N*H*-, ^3^J_H-H_ = 8.7 Hz), 6.82–7.30 (m, 7 H, H-4, H-3′, H-5′, H-2, H-6, H-7, H-8), 7.43 (d, 2 H, ^3^J_H-H_ = 8.5 Hz, H-2′, H-6′); ^13^C-NMR (300 MHz, CDCl_3_) δ(ppm): 170.7, 170.6, 154.1, 153.7, 151.4, 150.6, 139.1, 134.0, 129.2, 127.3, 116.3, 114.2, 82.1, 58.6, 38.2, 27.9, 25.1, 15.3, 11.6. ESI-MS (ion trap): m/z 868 [M+H]^+^. HRMS (ESI+): *m/z* 868.4966 [M+H]^+^, calcd for C_47_H_70_N_3_O_12_: 868.4959.

#### (E)-di-tert-butyl 2,2′-((((5-(4-(((1,3-di-tert-butoxy-1-oxobutan-2-yl)carbamoyl)oxy)styryl)-1,3-phenylene)bis(oxy))bis(carbonyl))bis(azanediyl))bis(3-(tert-butoxy)butanoate) (**4c**)

Purified by flash chromatography using DCM:ethyl ether = 95:5 as eluent. 58% yield as a colourless oil. ^1^H-NMR (250 MHz, CDCl_3_) δ (ppm): 1.15 (s, 27 H, 9 × C-*CH*_*3*_), 1.23 (d, 9 H, 3 × CH-*CH*_*3*_, ^3^J_H-H_ = 6.2 Hz), 1.45 (s, 27 H, 9 × C-*CH*_*3*_), 4.04–4.25 (m, 6 H, 3 × CH_3_-*CH* and 3 × NH-*CH*), 5.78 (d, 3 H, 3 ×-N*H*-, ^3^J_H-H_ = 9.5 Hz), 6.85–7.30 (m, 7 H, H-4, H-3′, H-5′, H-2, H-6, H-7, H-8), 7.43 (d, 2 H, ^3^J_H-H_ = 8.5 Hz, H-2′, H-6′); ^13^C-NMR (62.9 MHz, CDCl_3_) δ (ppm): 169.6, 154.7, 154.3, 151.4, 150.5, 139.0, 133.9, 129.1, 127.3, 127.1, 121.6, 116.2, 114.1, 81.9, 81.8, 73.7, 66.9, 60.3, 28.5, 27.9, 20.8. ESI-MS (ion trap): m/z 1001 [M+H]^+^. HRMS (ESI+): *m/z* 1000.5754 [M+H]^+^, calcd for C_53_H_82_N_3_O_15_: 1000.5746.

#### (E)-di-tert-butyl 2,2′-((((5-(4-(((1-(tert-butoxy)-1-oxo-3-phenylpropan-2-yl)carbamoyl)oxy)styryl)-1,3-phenylene)bis(oxy))bis(carbonyl))bis(azanediyl))bis(3-phenylpropanoate) (**5c**)

Purified by flash chromatography using Hexane:Diethyl ether = 7:3 as eluent until the exit of 4-nitrophenol from the column, and Hexane:Diethyl ether:EtOAc = 5:3.5:1.5 thereafter. 57% yield as a colourless oil. ^1^H-NMR (250 MHz, CDCl_3_) δ (ppm): 1.41 (s, 27 H, 3 ×-C-C*H*_*3*_), 3.02–3.30 (m, 6 H, 3 × Ph-C*H*_*2*_), 4.16 (q, 1 H, NH-C*H*-CH_2_, ^3^J_H-H_ = 7.1 Hz), 4.64 (q, 2 H, 2 × NH-C*H*-CH_2_, ^3^J_H-H_ = 6.2 Hz), 5.79 (m, 3 H, 3 ×-N*H*-), 6.81–7.56 (m, 24 H, Ar-*H*); ^13^C-NMR (62.9 MHz, CDCl_3_) δ (ppm): 170.6, 154.0, 153.7, 151.6, 150.8, 139.4, 136.2, 128.8, 128.7, 127.7, 127.3, 122.0, 116.7, 114.5, 82.8, 55.6, 38.6, 28.2. ESI-MS (ion trap): m/z 970 [M+H]^+^. HRMS (ESI+): *m/z* 970.4499 [M+H]^+^, calcd for C_56_H_64_N_3_O_12_: 970.4490

#### General procedure for the tert-butyl ester deprotection in resveratrol-amino acid carbamoyl conjugates (**2–5**)

The t-butyl protected resveratrol-amino acid carbamoyl conjugate (**2c–5c**) (1.12 mmol) was dissolved in 10 mL of DCM at 0 °C. 500 μL of TIPS and 10 mL of TFA were then added and the reaction mixture was stirred at room temperature under nitrogen overnight. The solvent and residual TFA were removed under reduced pressure. The residue was triturated with toluene (3 × 5 mL), and the solvent was removed under reduced pressure. The product was purified by preparative reverse phase HPLC.

#### (E)-2,2′-((((5-(4-(((1-carboxy-3-methylbutyl)carbamoyl)oxy)styryl)-1,3-phenylene)bis(oxy))bis(carbonyl))bis(azanediyl))bis(4-methylpentanoic acid) (**2**)

Purified using a reverse-phase preparative HPLC column (ACE 5AQ 150 mm×21.2 mm from 7.5% to 38% of ACN in water in 14.5 minutes). 89% yield as a white powder after freeze-drying. ^1^H-NMR (250 MHz, DMSO-d_6_) δ (ppm): 0.89–0.94 (m, 18 H, 3 ×-CH(C*H*_*3*_)_2_), 1.45–1.81 (m, 9 H, 3 ×-CH-C*H*_*2*_- and 3 × (CH_3_)_2_C*H*-), 3.98–4.07 (m, 3 H, -NH-C*H*-), 6.76 (t, 1 H, ^4^J_H-H_ = 2 Hz, H-4), 7.10–7.38 (m, 6 H, H-3′, H-5′, H-2, H-6, H-7, H-8), 7.63 (d, 2 H, H-2′, H-6′, ^3^J_H-H_ = 8.7 Hz), 8.12–8.21 (m, 3 H, 3 ×-N*H*-); ^13^C-NMR (250 MHz, DMSO-d_6_) δ(ppm): 174.1, 174.0, 154.4, 154.2, 151.6, 150.7, 139.1, 133.7, 129.3, 127.6, 126.8, 121.9, 116.3, 114.4, 52.5, 39.6, 24.4, 23.0, 21.2. ESI-MS (ion trap): m/z 700 [M+H]^+^. HRMS (ESI+): *m/z* 700.3092 [M+H]^+^, calcd for C_35_H_46_N_3_O_12_: 700.3081.

#### (E)-2,2′-((((5-(4-(((1-carboxy-2-methylbutyl)carbamoyl)oxy)styryl)-1,3-phenylene)bis(oxy))bis(carbonyl))bis(azanediyl))bis(3-methylpentanoic acid) (**3**)

Purified using a reverse-phase preparative HPLC column (ACE 5AQ 150 mm×21.2 mm from 40% to 70% of ACN in water in 11 minutes). 93% yield as a white powder after freeze-drying. ^1^H-NMR (300 MHz, DMSO) δ (ppm): 0.86–0.95 (m, 18H, 3 ×-CH_2_-C*H*_*3*_ and 3 ×-CH-*CH*_*3*_), 1.21–1.55 (m, 6H, 3 ×-CH-C*H*_*2*_), 1.80–1.91 (m, 3 H, -CH-C*H*-CH_3_), 3.89–4.13 (m, 3 H, 3 × NH-*CH*-CH), 6.77 (t, 1 H, ^4^J_H-H_ = 2 Hz, H-4), 7.06–7.37 (m, 6 H, H-3′, H-5′, H-2, H-6, H-7, H-8), 7.63 (d, 2 H, H-2′, H-6′, ^3^J_H-H_ = 8.7 Hz), 8.04 (d, 1 H, -N*H*-, ^3^J_H-H_ = 8.4 Hz), 8.10 (d, 2 H, 2 ×-N*H*-, ^3^J_H-H_ = 8.4 Hz); ^13^C-NMR (300 MHz, DMSO) δ(ppm): 175.1, 175.0, 156.7, 156.5, 153.8, 152.9, 141.3, 135.8, 131.5, 129.8, 124.1, 118.4, 97.3, 61.0, 38.3, 26.9, 17.8, 13.5. ESI-MS (ion trap): m/z 700 [M+H]^+^. HRMS (ESI+): *m/z* 700.3075 [M+H]^+^, calcd for C_35_H_46_N_3_O_12_: 700.3081.

#### (E)-2,2′-((((5-(4-(((1-carboxy-2-hydroxypropyl)carbamoyl)oxy)styryl)-1,3-phenylene)bis(oxy))bis(carbonyl))bis(azanediyl))bis(3-hydroxybutanoic acid) (**4**)

Purified using a reverse-phase preparative HPLC column (ACE 5AQ 150 mm×21.2 mm from 7.5% to 38% of ACN in water in 14.5 minutes). 91% yield as a white powder after freeze-drying. ^1^H-NMR (250 MHz, DMSO-d_6_) δ (ppm): 1.17–1.19 (m, 9 H, 3 ×-CH-C*H*_*3*_), 4.00–4.23 (m, 6 H, 3 × CH_3_-C*H*- and 3 × NH-C*H*-), 6.80 (t, 1 H, ^4^J_H-H_ = 2 Hz, H-4), 7.01–7.39 (m, 6 H, H-3′, H-5′, H-2, H-6, H-7, H-8), 7.61–7.72 (m, 5 H, 3 ×-N*H*- and H-2′, H-6′); ^13^C-NMR (250 MHz, DMSO-d_6_) δ(ppm):172.1, 172.1, 154.8, 154.6, 151.7, 150.8, 139.2, 133.8, 129.4, 127.7, 126.9, 122.0, 116.4, 114.6, 66.5, 60.4, 48.7, 20.5. ESI-MS (ion trap): m/z 664 [M+H]^+^. HRMS (ESI+): *m/z* 664.1993 [M+H]^+^, calcd for C_29_H_34_N_3_O_12_: 664.1990.

#### (E)-2,2′-((((5-(4-(((1-carboxy-2-phenylethyl)carbamoyl)oxy)styryl)-1,3-phenylene)bis(oxy))bis(carbonyl))bis(azanediyl))bis(3-phenylpropanoic acid) (**5**)

Purified using a reverse-phase preparative HPLC column (ACE 5AQ 150 mm × 21.2 mm from 35% to 65% of ACN in water in 13 minutes). 91% yield as a white powder after freeze-drying. 1H-NMR (250 MHz, DMSO-d_6_) δ (ppm): 2.86–3.19 (m, 6 H, 3 × Ph-C*H*_*2*_), 4.21–4.30 (m, 3H, 3 ×-NH-C*H*-CH_2_), 6.47 (t, 1 H, ^4^J_H-H_ = 2 Hz, H-4), 6.91–7.33 (m, 21 H, Ar-*H* and H-3′, H-5′, H-2, H-6, H-7, H-8), 7.58 (d, 2 H, H-2′, H-6′, ^3^J_H-H_ = 8.7 Hz), 8.20–8.29 (m, 3H, 3 ×-N*H*-); 13C-NMR (62.9 MHz, DMSO-d_6_) δ (ppm): 173.0, 154.3, 154.0, 151.5, 150.7, 139.1, 137.8, 137.8, 133.6, 129.3, 128.3, 127.6, 126.6, 121.8, 116.1, 114.2, 55.8, 55.0, 36.6; ESI-MS (ion trap): m/z 802 [M+H]^+^. HRMS (ESI+): *m/z* 802.2604 [M+H]^+^, calcd for C_44_H_40_N_3_O_12_: 802.2612.

### Stability under physiologically relevant conditions

The stability of all new compounds was tested in aqueous media imitating gastric (0.1 N HCl, NormaFix) and intestinal (0.1 M PBS buffer, pH 6.8) conditions. A 5 μM solution of the compound was prepared from a 5 mM stock solution in DMSO, and incubated at 37 °C for 24 hours; samples withdrawn at different times were analysed by HPLC-UV. Hydrolysis products were identified by HPLC/ESI-MS analysis of selected samples. Non-linear curve fitting was performed using Origin 8.0 data analysis software, using the equations described in^55^,^61^.

### Stability in rat whole blood

Rats were anesthetised and blood was withdrawn from the jugular vein, and transferred into tubes containing heparin. Blood samples (1 mL) were spiked with 5 μM compound (dilution from a 5 mM stock solution in DMSO), and incubated at 37 °C for 4 hours (the maximum period allowed by blood stability). Aliquots were taken after 10 min, 30 min, 1 h, 2 h and 4 h and treated as described below (blood sample treatment and analysis). Cleared blood samples were finally subjected to HPLC-UV analysis.

### Blood sample treatment and analysis

Before starting the treatment, 4,4′-dihydroxybiphenyl was added as internal standard to a carefully measured blood volume (25 μM final concentration). 0.1 vol of 0.6 M TEA (pH 8.0) and 4 vol of MeOH were then added to the blood samples, which were then sonicated (2 min) and centrifuged (12,000 g, 7 min, 4 °C). The supernatant was finally collected and stored at −20 °C. Before analysis, MeOH was evaporated off at room temperature using a Univapo 150H (UniEquip) vacuum concentrator centrifuge, and up to 40 μL of ACN were added to precipitate residual proteins. After centrifugation (12,000 g, 5 min, 4 °C), cleared samples were directly subjected to HPLC-UV analysis. Metabolites and hydrolysis products were identified by comparison of chromatographic retention time with true samples or by HPLC/ESI-MS analysis.

Internal standard recovery from the treatment used to extract resveratrol-AA derivatives was 91.8 ± 10.7%. The recovery of **3**, expressed as ratio to the recovery of internal standard, was 0.633 ± 0.088. Recoveries of partially protected (disubstituted) derivatives (Fig. 7) were assumed to be the same as those of the corresponding fully substituted prodrug. Knowledge/assumption of these ratios allowed us to determine the unknown amount of analyte in a blood sample by measuring the recovery of internal standard^57^.

Since sample treatment includes an evaporation/concentration step, LOD and LOQ were determined relatively to the analytical part of the method (HPLC/UV analysis). The prodrugs have the same absorption coefficient of resveratrol itself within experimental error; LOD and LOQ were thus the same of resveratrol (i.e., 0.04 and 0.12 μM, respectively^58^), and quantification of the analytes in blood samples was done using the same calibration curve of resveratrol (y = 5.3085 x), taking into account the recovery ratio.^57^

### Pharmacokinetics studies

Derivatives **2–5** were administered to overnight-fasted male Wistar rats from the facility of the Department of Biomedical Sciences, University of Padova, as a single intragastric dose (88 μmol/Kg, dissolved in 250 μl DMSO). Blood samples were obtained by the tail bleeding technique: before drug administration, rats were anesthetised with isoflurane and the tip of the tail was cut off; blood samples (80–100 μL each) were then taken from the tail tip at different time points after drug administration. Blood was collected in heparinised tubes, kept in ice and treated as described above within 10 min.

All experiments involving animals were approved by the University of Padova Ethical Committee for Experimentation on Animals (CEASA) and performed with the supervision of the University Central Veterinary Service, in compliance with Italian Law DL 116/92, embodying UE Directive 86/609.

## Additional Information

**How to cite this article**: Mattarei, A. *et al.* Amino Acid Carbamates As Prodrugs Of Resveratrol. *Sci. Rep.* **5**, 15216; doi: 10.1038/srep15216 (2015).

## Figures and Tables

**Figure 1:**
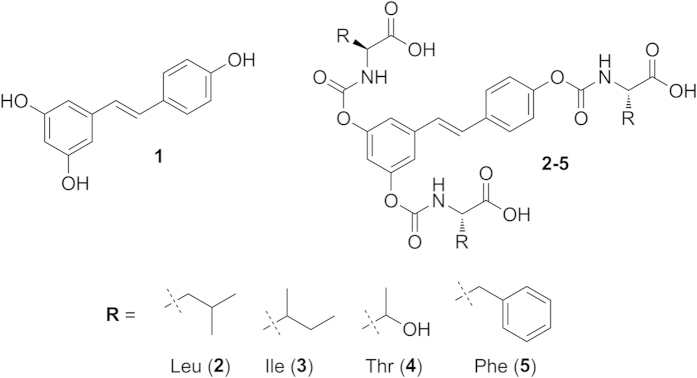
Resveratrol (1) and new amino acid substituted prodrugs (2–5).

**Table 1:** Observed pseudo first-order rate constants for hydrolysis of resveratrol derivatives 2–5 in aqueous PBS 0.1 M at pH 6.8 and 37 °C and in rat blood.

Derivative	t_1/2_(h)	PBS 0.1 M, pH 6.8, 37 °C			Blood			
		10^2^ * k_III_ (h^−1^)	10^2^ * k_II_ (h^−1^)	10^2^ * k_I_ (h^−1^)	t_1/2_(h)	k_III_ (h^−1^)	k_II_ (h^−1^)	k_I_ (h^−1^)
2	>24	1.98 ± 0.03	0.9 ± 0.2	*	1	0.57 ± 0.06	ND#	ND#
3	>24	2.21 ± 0.02	0.4 ± 0.4	*	1	0.63 ± 0.04	0.12 ± 0.03	0.4 ± 0.1
4	15	5.30 ± 0.08	1.4 ± 0.1	0.6 ± 0.1	0,17	3.9 ± 0.2	0.057 ± 0.006	0.45 ± 0.09
5	17	4.25 ± 0.02	1.36 ± 0.04	0.2 ± 0.1	0,3	1.86 ± 0.06	0.056 ± 0.007	0.09 ± 0.02

Values ± standard error are reported as obtained from the fit of all the available data.

^*^k_I_ not determined; monosubstituted derivative detected only at the latest time point.

^#^k_II_ and k_I_ not determined because of co-elution of di- and mono-substituted derivatives with matrix background interfering peaks.
